# The Process of Creating Integrated Home Care in Lithuania: from Idea to Reality

**DOI:** 10.5334/ijic.2509

**Published:** 2016-08-19

**Authors:** Ramunė Jurkuvienė, Lina Danusevičienė, Rūta Butkevičienė, Indrė Gajdosikienė

**Affiliations:** 1 Department of Social Sciences and Humanities, Faculty of Public Health, Medical Academy, Lithuanian University of Health Sciences, Lithuania; 2 Department of Nursing and Care, Faculty of Nursing, Lithuanian University of Health Sciences, Lithuania; 3 Department of Social Work, Faculty of Philosophy, Vilnius University, Lithuania

**Keywords:** integrated home care, social innovation, dialogue culture, post-soviet context

## Abstract

**Background:**

The article presents an analysis of the formulation and implementation of a social innovation: integrated home care (IHC) in post-soviet Lithuania. From 1998 a series of top-down orders to implement IHC were issued, however, home nursing did not start. In 2011 the Ministry of Social Security and Labour began a process to develop integrated home care using new, collaborative processes. The result was 21 pilot projects with well-conceptualized IHC services.

**Method:**

Using data from focus groups, interviews, and recorded observations, the research team systematically documented the innovation process, including themes and deviations, employing Smale’s Innovation Trinity framework to organize the larger picture.

**Results:**

In the Lithuanian post-totalitarian context, top-down communication was found to be prevalent. Not only IHC, but also openness to change and dialogue at high levels were innovations. Patient-centered practice at local levels could only occur when a new attitude of mind was reached through dialogue with officials at higher levels and between peers.

**Conclusions:**

The enactment, rather than the mask of dialogue, participatory program development were critical in the success of IHC innovation. This is difficult to achieve in the light of antiquated public bureaucracies, but in this case, the Ministry team, rather than avoiding the expectation of top-down communication, made it into an asset through promotion of collaboration.

## Introduction

The aims of our study were to document and analyse the innovations of creating and implementing integrated home care (IHC) in the context of rigid medical ideology and bureaucracy in post-soviet Lithuania. Learning what made the IHC innovation successful when the previous attempts to implement it failed can have positive implications for other settings with these characteristics.

After independence from the Soviet Union in 1990, Lithuania inherited government structures that included rigidly separated health and social security bureaucracies. The health care system depended on small, local hospitals [1]. If one was “sick,” one went to the hospital; if one stayed at home, one was “well” by definition. No reality between these was officially recognized. Most of the small hospitals were replaced by larger, consolidated ones that were thought to be more efficient [2]. Social care existed in the soviet society, but it consisted primarily of institutional care and control for specific categories of people [3]. Structural reforms were attempted in both health and social care ministries, but the boundary between them was seldom permeated. Each of the repeatedly changing political administrations usually discontinued initiatives from the previous one. Almost no home care services were started to replace the small hospitals’ role in care, although the Ministry of Social Security and Labour (MSSL) began very limited assisted care at home, focusing on lonely elderly people. While primary health care inherited rigid practices and structures, the seeds of a new social care system were being sewn.

Employment of trained personnel from the new profession of social work was mandated in law. The social service centres were staffed with social workers and assistants who worked in communities and homes. Many social care officers understood that health needs were not being addressed, as the nurses from the primary care centres would visit a patient at most once a month for narrowly medical purposes. Often even this limited care was not provided. The need for more adequate medical care, and for integrated home care (IHC) that would combine medical and social services became clear to many working on the social care side of the picture. But how could IHC be created? In 1998 the Minister of Social Security and Labour issued an order to implement home care services at the local level, with the emphasis on integrated care [4]. Virtually nothing resulted. There were no nurses in the social care system. There was no interest in community care from medical officials because payment would be inadequate and partly because of a lack of understanding. In 2007 the Minister of Health and the Minister of Social Security and Labour issued a joint order to establish collaborative home care services [5]. Again, IHC did not result. The response was similar to the process in soviet times: authoritarian orders from above frequently were not implemented in reality if there was no consequence for inaction.

Things began to change in 2012. New personnel in the MSSL introduced the idea of pilot IHC projects designed with input from the local staff, which could take advantage of external funding opportunities. The Minister for Social Security and Labour issued an order “Regarding the Approval of the Program of Integrated Care Development” [6]. The process behind this order was something new: it was a result of a year of intensive discussions of a more professionally oriented administration of the MSSL with the 21 (out of 60) municipalities’ officials. They expressed their interest in creating pilot IHC projects. The idea was to supplement the existing social care services with newly created teams of nurses, their assistants, and kinesiotherapists. The creators of the IHC model expected that health professionals would work together with social care providers to establish mobile teams of integrated care to provide seamless, patient-centered services at home. Specific organizational forms and practices were detailed, and in 2013 municipalities began their projects with money secured from the European Social Fund.

## The Study

The researchers’ fundamental assumption was that IHC in Lithuania was a social innovation and could be studied as such. This assumption was based on team members’ familiarity with the lack of IHC services. The assumption was strengthened when their literature review yielded no relevant citations. The European literature confirmed the lack of development of IHC in Eastern and Central Europe, including Lithuania. In 2011 Genet et al. [7] published the results of a systematic literature review of home care in Europe, citing only descriptive studies from Poland [8], Slovenia [9] and Czech Republic [10] on Eastern side. While they found 5133 articles related to home care, no studies of any kind from Lithuania were found in their search of eight established academic databases, supporting the assumption that IHC in Lithuania was indeed an innovation.

This study is a description and analysis of the innovation and how it came about, as experienced by the people who were directly involved. The study intent was to provide a data-driven, reality based assessment of a new application of IHC, to gain understanding about the process of creating IHC projects and how this time it was reached that IHC teams started to visit persons with the need of long-term care at home, and started to provide care. The study can be thought of as action research in that the study’s research team had followed, and to different degrees participated in the development of, the IHC innovations. As discussed by Argyris and Schön, “Action research takes its cues – its questions, puzzles, and problems – from the perceptions of practitioners within particular, local contexts. …’[11].

In this qualitative study, it is important to document and bracket the positions of the researchers in the compact world of Lithuanian health and social care. One member of the research team held a key appointment at the MSSL at the time the integrated home care ideas were being developed. This situation is typical in action research. Instead of being an impediment to accurate data collection, analysis, and interpretation, this affiliation and involvement in the work was framed as an asset in the case study of how something developed. The researcher’s knowledge of the innovation’s intent and structure made it possible to focus on key elements of the innovation, such as the role of the local social care authorities in developing the new model that the Ministry advocated. These study characteristics clearly provide one perspective, and thus it can serve as a basis for additional studies, particularly systematic studies of service effectiveness, which were beyond the scope of the current research.

## Theory and methods

The study emphasizes understanding the process of creating new and very different services in the post-soviet context, following Stake’s principles of case study research on a “unique and bounded system” [12, p. 121] which is “real-life grounded and related to contemporary events”. The case study design allowed the team to concentrate on the experiences and perceptions of the individuals involved in the innovation as well as the larger social process. A cycle of induction (looking for patterns in the raw data) and deduction (looking for consistency and generalizability in identified patterns) was deliberately applied. The researchers’ understanding of the social and political contexts could thereby also be incorporated in the analysis [13, p. 135].

The researchers began with the conceptual framework of understanding of strategic change as holistically as possible [13, p. 26]. While not derived into specific a priori hypotheses, key elements framed the research process: reasons for the change, the process of change, and the content of change. These elements guided much of the questioning and observing that occurred during data collection and, to some extent, analysis.

The primary participant-researcher who was active at the MSSL had an academic background in innovation in health and social care. The research interview with her by another team member revealed that she had relied from the beginning on ideas based on Smale’s “innovation trinity”: context, the specific content of the innovation, and the interactions of the key people involved [14,15]. The tacit theoretical formulation of the trinity of context, content, and key people’s interactions that guided the project became explicit through the iterative processes of data collection and analysis. As the analysis progressed the entire team found that Smale’s ideas on innovation were relevant and valuable in understanding the process that was being uncovered and documented. For understanding the change the idea of “dialogue culture” became increasingly important, used in the sense of Freire as a cooperative activity of equal participants and dealing with naming and transforming reality [16, pp. 63–65]. As we develop the ideas below, it will be seen that in the study’s social and historical context, developing ideas collaboratively was not only new, it was extremely powerful and effective.

*Data sources and collection*. A total of 106 service users, administrators, other officials, nurses and nurse’s assistants, social workers and social work assistants contributed data to the project. The data was obtained through interviews and focus groups. Table 1 and Table 2, respectively, summarize the profiles of professional and service user groups and the types of data collection that was used. Individual interviews were used in the cases of administrators and service users, since they worked relatively independently from the service providers and were relatively office-bound. Focus groups were used with the professionals owing to their availability and their work together on teams. Each discipline was grouped so as to gain on what had happened and how it was working. Consistent with the study team’s guiding idea that context was important, professional groups were used to discover any contrasts in perceptions related to different contextual backgrounds.

**Table 1:** Description, number and occupation/status of professional IHC project informants from municipalities, and data collection methods.

**Informants**			
*Total number of interviews and focus groups n* = *44*			

**IHC service providers**			
*Total number n* = 106*			

***General group description (total number)***	***Occupation***	**n**	***Data collection methods***

Administrators and officials involved in implementation IHC (n = 20)	Administrators	12	In-depth individual interviews
	Officials	8	

Multidisciplinary teams from 10 regionally diverse pilot municipalities (n = 51*)	Social workers	12	10 focus groups
	Nurses	11	
	Physiotherapists	7	
	Social worker assistants	11	
	Nurse assistants	10	

Front-line staff from 20 municipalities according disciplines (n = 59*)	Social workers	20	4 focus groups
	Nurses	19	4 focus groups
	Social worker assistants	9	4 focus groups
	Nurse assistants	11	

*24 people participated both in the multidisciplinary and in the across the municipalities focus groups.

**Table 2:** Description of service receivers providing in-depth interviews.

**Informants**			
*Total number of interviews n* = *34*			

**IHC service receivers**			
*Total number n* = *34*			

***General group description***			***n***

Family caregivers (n = 14)	Social status	Daughter/daughter-in-law	9
		Mothers/fathers	2
		Spouses	3
	Gender	Female	12
		Male	2

Care receivers-patients (n = 20)	Age	50–64	2
		65–80	18
	Gender	Female	18
		Male	2

The data was collected in several steps after the IHC innovations were in place and operating. At first, an administrator of a regional polyclinic and another administrator of a small local primary health unit were interviewed in order to gain understanding about the medical context of home nursing and why it had not been provided. Next, the stakeholders in IHC project development were interviewed. Two officials of the MSSL who were in charge of the IHC program participated. Ten IHC teams were purposively selected to represent the diverse settings in which IHC services were offered. Settings included social service centres, a polyclinic, a private health clinic, and residential units for the elderly. The teams’ administrators were interviewed. Patients and their families from these communities were interviewed, sometimes independently and sometimes together. Front line staff provided data primarily in focus groups that were conducted according to discipline, with members from all pilot municipalities. The focus groups provided informants the chance to share their experiences, opinions and insights and for researchers to obtain deeper and richer information [17,18]. The focus groups took place in the municipality offices and lasted 90–120 min. each. All interviews were tape-recorded with the informants’ consent. The interviews were semi-structured and carried out using an interview guide consisting of questions about the participants’ experience with the creation and functioning of IHC. The guide was constructed on the basic ideas of Pettigrew and Whipp: conceptualizing the project, clarifying the reasons *(why)* that it was created, describing the process of implementation of IHC, and difficulties that were experienced (*content and process*). The guide for service users encouraged them to tell their stories about their situation before and with IHC (*content*) [13, p. 26].

*Data analysis*. Beginning in April 2014 the team met regularly to present the information that each member had collected, and to discuss it in light of other team members’ findings and understandings. This was an evolutionary process in which themes became not only more and more repetitive, but also clearer over a year. In the process of data analysis, a team member suggested to use Smale’s “Innovation Trinity” framework, with the dimensions of understanding the context, analysis of the nature of the innovation, and identifying the key people. After additional discussion and attempts to find data and themes that might be inconsistent with the framework, and thus call its use into question, the trinity was adopted as a structure for data analysis.

## Findings

Smale begins his framework by considering the culture of the organization and society that often go unexamined. Thus, if the innovation trinity was present from the start of the project in the form of a tacit working hypothesis about what would be important in IHC innovations, the main finding is that central elements of each part of the trinity were indeed observed. This is not surprising, given the interacting elements of the action research approach that was used, but it is no less useful in understanding and promoting innovation in a society transitioning from authoritarianism to more participatory forms of social organization. Thus, the findings are organized along the three dimensions that Smale presented. First, the post-soviet context of IHC creation is reflected in section 1, which often brings innovators to helplessness. Most of this information is based on accounts of the way that things were before the innovation process began. Three basic, problematic contextual elements within the context were identified. Second, an analysis of IHC as a social innovation as experienced and understood by informants is presented, corresponding to the second element of the Smale formulation. This second section describes the development of the idea of IHC as a patient-centred service including the processes of promoting dialogue as an innovation and implementing the ideas in practice. Section 3 presents results about the characteristics of and negotiations with the key participants.

## The overall context: the lingering effects of soviet systems and mindsets

An innovation is not necessarily a new or unique happening. An innovation is a solution to problems that is new for the specific context [15, p. 113]. For an innovation, in this case, IHC, to be accepted, it is important for the innovators to realize the contexts of those who would implement and adapt the innovative idea, “to identify how key dimensions of the human and wider environment can be used to promote desired change” [15, p. 117]. The research team grew up in Lithuania when it was an occupied part of the Soviet Union. They were highly aware of the soviet style and practice of bureaucracy and they had graduate degrees in social work taught primarily by leading Western academics. They were well equipped to understand the fundamental historical context of the study, and unlike many of their fellow former soviet citizens, they knew alternative ways of achieving things.

The revolutionary changes resulting in independence in 1990 had little effect on the way that senior bureaucrats thought and thus the way that organizations functioned. Primary healthcare, including nursing at home, was declared Lithuania’s priority in 1991, very soon after independence [19]. As with many such early reform declarations, little or nothing resulted in the way of service provision, training, or research. Moreover, a series of strategic papers advocating IHC were issued starting from 1998. Health care officials and social care officials, at least, logically would have been expected to collaborate at this point. However, the reality was different. The study revealed a complex net of characteristics prohibiting collaboration, innovations, and realistic problem-solving. These all were elements of the mosaic of post-totalitarian Lithuania. Repeatedly, impulses toward reform and better treatment of citizens met resistance, both blunt and subtle, most often reflected in attitudes of mind formed in the past, in the maintenance of control as a higher value than service, and in isolation from the new ideas and practices developing outside the field of view of those in power. Interestingly, many younger people demonstrated the same characteristics of rigidity that they learned during the tumultuous period of chaos and transition, perhaps clinging to them as natural and secure because of the turmoil.

### Context: “Laws are not discussed – they are obeyed”

The phrase was often used by the research participants to reflect the usual requirement from above to obey expectations, rules, or regulations issued from higher up the bureaucracy. Several research participants used variations of the phrase “laws are not discussed – they are obeyed”. They expressed the hard and fast requirement to obey rules and to conform to regulations, and expectations that came from above. Typically, these role expectations were literal applications of laws and orders. Informants frequently spoke of this role expectation with a flavour of helplessness. Unfortunately, when the hierarchy sees the top-down directives as the main way of developing services and does not allow genuine feedback, the decisions frequently take the form of illogical orders and instructions that do not serve the patient. An administrator who tried to obtain a nursing and kinesiotherapy licence for their social agency in the IHC project provided this example:

“We had to install a bed for kinesiotherapy in the office, although we were telling [the controlling person] we will provide kinesiotherapy at people’s home. We were told, “such are the requirements, and we will not lower the hurdle because of you.” (Administrator of IHC, site 5)

Inflexibility, lack of caring dialogue and exhibition of power were leading to waste of resources. In this example a massage bed that would not ever be used was approved through the bureaucratic structure. The only purpose of the decision was to reinforce top-down control.

Furthermore, the strategy to survive in the conditions of senseless requirements had a built-in dysfunctional reciprocal practice. The widespread “normal” practice continued to be reporting compliance, regardless of the reality. For example, when the requirements to provide home care appeared, the healthcare administrators treated them as impossible to implement with their resources. While their healthcare agencies did not provide any help at home, they reported that they had provided some. The control of service delivery is strictly hierarchical and formalised [2], therefore the reports, rather than reflecting the reality, showed what people “above” would expect to see.

“In our healthcare system everything is very clear: we do only what is ordered from “above” in a written form. Even in the cases when in reality we are not able to perform the orders from above, at least in papers and documents we have to show that everything is performed in a perfect way. The bosses are checking us only according to the papers, anyway. And paper, as you know, is able to bear anything…” (Manager 1 of primary healthcare unit, site 6)

Meanwhile the patients and their family caregivers did not know that it should have been possible to get at least some medical services at home, although they had an extreme need for it. Access to services was encumbered by bureaucratic requirements, and chronically ill people or their overburdened family members had serious difficulty trying to fulfil them. Thus some of the people who were most in need of help from both systems (health and of social care) because of being sick and single, were not getting care since they were unable to collect all the requested documents. There were no statistics concerning these cases. Both service providers and service receivers felt helpless and trapped by the legislation that did not fit the reality. In some cases available money was not used for home care owing to a lack of interest in IHC on the part of primary care providers and nurses. There were no explanations from the Ministry of Health about why funds could not be used for home care, the very purpose for which they were intended. The professionals treated such situations as social forces that they were powerless to change. Meanwhile the Ministry of Health acted as if the directives for home nursing were being followed, but not very fully. No effort was made to find out what was actually going on in the field, and all efforts to provide feedback were bluntly resisted. The fact that the money for home care services was not being used fully was interpreted as a lack of need for home care.

Such formalized top-down communication creates a cycle of pseudo-reality out of several interdependent bogus realities. At its core, documented, though fake, implementation of rules and requirements is required at the point of service delivery; these fake documentations are received at the top, leading to new judgments and policies that can have fatal consequences for patients and their families. People needing home care were left with needs, available resources did not match the needs. This led to situations like the one reported by a family member who could not get adequate nursing home care for her mother:

“What kind of bedsores we had! I don’t know, it seems we were turning her [chronically ill mother] over and everything, but… We didn’t know how to treat [them]. […] The wounds became wet, really deep, even to the bone…” (Daughter, site 21)

In the study, we learned that there were a number of practitioners who were eager to change this way of thinking and working, but that *contextual*, organizational conditions had held them back.

### Context: Horizontal ties are not valued (“the order to collaborate is enough”)

When people in an illogical environment are preoccupied with the strategies of pretending, it is very difficult for them to envision and achieve goals that are important to themselves, their clients, or the whole. Teamwork is secondary to survival. With the internalized habit of complying with authority, horizontal relationships based on respect are hard to form, and eventually are not valued. In this context the patient-centred approach was unlikely to take root.

In response to the 2007 order to establish IHC the relationship of social care and healthcare sectors was reciprocal but not equivalent: the initiative for collaborative relationships almost always came from the social care sector. Based on the informants who discussed this, it may be that this was due not only to the rigidity of the medical system, but also to the ideas of social workers whose recent education was influenced by Western ideas about integration and collaboration.

When the representatives of the social care system were not satisfied with the lack of home care and tried to discuss it with the representatives of the Ministry of Health, they were met with resistance. Health policy personnel

*“were saying, that there is no need to change anything, that the order of collaboration between sectors, signed by both Ministers [for Health, and for Social Security and Labour], is enough” (2nd informant of macro level)*.

Based on this order [5], some local health and social care officials had agreements to provide collaborative IHC. However, the only expression of collaboration was exchanging quarterly paper reports about the services provided. When the horizontal relationships were not valued or enacted the word “collaboration” took on a weird meaning.

“We [social workers] kept asking that their nurses visit the people who were receiving social home care services, at least once a month. We had to ask, “Please, visit.” But they didn’t come.” (Social worker, administrator of IHC project, site 4)

“They [social workers] are collaborating with us, but we [the primary healthcare unit] are not [collaborating with them].” (Head of primary healthcare centre, site 5)

In summary, in the context of authoritarian relationships, the vertical ties and power relationships were perceived by many informants as necessary for survival and the importance of horizontal ties was not widely understood. Thus the organizational and cultural context all but eliminated the possibility of true dialogue, characterized by equal treatment, giving and receiving feedback, reacting to a changing situation, caring listening to the other one. Many of the actors had never known any other way.

### Context: Uniformity versus creativity

Requesting and expecting uniformity across service delivery organizations and even clients was another post-soviet effect. Uniformity is one of the ways to establish hierarchical control, which is still considered one of the most important functions of the public sector. Creativity and initiative can be a threat to the stability of the system, therefore innovators were typically forbidden and punished rather than encouraged or rewarded. One of the most important threats to centralized, uniform control was found to be the employment of nurses by the social care agencies.

“Already in the year 2000 during the creation of our social service department, we founded a subdivision of home care services. […] At that time we were providing service very close to the idea of current integrated team-based model. But after a few years we had an audit. They [audit] explained to us that we do not have the right to provide home nursing as we as an agency do not have a license [for health care services]. Our nurses had licenses, but for them as specialists [in the medical hierarchy]. At that time there was no system that social care unit could get the license. The audit conclusions were presented [to the municipality], and we had to terminate our nursing services. Only social home care services were left. We had to fire the community nurses.” (Manager of social service centre, site 4)

When initiative is not allowed or is punished, people learn not to take risks. They may try to act exactly as ordered, or at least to appear to, and thus be less likely to attract unnecessary attention. Moreover, when uniformity is requested, the interest of how others develop different solutions, and learning through horizontal ties become meaningless. Many workers participating in the innovation and the study had become accustomed to the uniformity of rules and limited services. Yet IHC depends on specific responses to the social and medical situation of each patient, often involving flexibility and creativity. The force of authoritarian uniformity made the implementation of IHC unlikely, and in fact served against even considering new IHC ideas.

Taking orders from above has another dimension. When an employee is told what to do and he or she does it, responsibility for the outcome is not in the hands of the employee. The internalised attitudes of passing all the responsibility to the top, not taking the horizontal relationships seriously, and expecting uniformity were summarized in the following opinion of a pilot municipality administrator about the discussions preparing the IHC program:

“We [people from municipalities,] can speak and discuss between ourselves as long and as much as we like. So what? The Ministry has to nevertheless state very clearly and specifically to us, how to work, and how services should be provided in the right way.” (Manager of social service centre, site 3)

When the decisions come from above without consultations about the local realities, and having common goals and looking for best solutions in collaboration is not the norm, the variety of opinions that may occur is not appreciated. Practitioners feel helpless about the situation, and they may lose their sense of caring. Many of the resulting relationships that were identified in the study were characterized by mistrust and isolation.

## The innovation: through dialogue to pilots of integrated home care

The specific new ideas and practices that constitute an innovation are the second element of Smale’s trinity. Part 2 of the findings describes the origin and unfolding of IHC as an innovation. The idea of starting IHC within the social care system was innovative for Lithuania. It offered the possibility of the first realistic patient-centred integrated help at home. However, not only the service concept, but also a process through which it had been born was an innovation. For the first time, the local officials at the municipalities were offered the opportunity to be involved in the search for solutions when creating a new service, which allowed paying attention to the realities of patients and practitioners, and fostered initiative and responsibility. The innovative aspects of the services that they implemented are summarized at the end of the section.

### The innovation: “Patient-centred means integrated”

In 2011 the Ministry of Social Security and Labour formed a new leadership team at the top. The leadership group was characterized by partnership and honest dialogue to address problems. This way of working was a drastic departure from the enduring bureaucratic status quo. A social worker with expertise in innovations and a background in medicine joined the group. The group immediately supported her idea of creating home care services that could meet the complex needs of chronically ill people and their caregivers. The innovators wanted to adapt the patient-centred approach to service delivery that they observed in Western countries, i.e. that services at home are provided according to the needs of the person and of the informal caregivers. The efforts to reach this goal together with the healthcare sector seemed to be fruitless. The apparent impasse with an uncooperative health system led to the new way of thinking that was crucial to the project. With the acceptance that the health authorities were almost certain not to change, the new idea took shape: to create integrated team-based home care services within the existing social care system, using the municipal net of social home care services for persons with the need of long-term care. For creating the services, the units of social care providers had to employ nursing specialists.

“We were trying to deal with the failures of the previous attempts, so [we wanted] that the new integrated care services would be coordinated and funded from one centre in a municipality, which would get the necessary money for it through the European Social Fund. Thus we would avoid the separation of social and healthcare sectors, office-like treatment, as well as constant miscommunication, because the social and nursing services would be provided by integrated care teams together.” (2nd informant of macro level)

The patient-centred idea that was behind the integrated care was new in Lithuanian practice. In many program sites it brought real change, enabled flexibility, team-work and real help for the patients.

The innovators understood that they were “invading” the territory of healthcare; however, they also clearly understood that their aim is to help people in a very important area, and they did not want to be left helpless because of the lack of collaboration on the health side. When the simple but drastic idea was presented to the people from municipalities, both resistance and advocacy flowered:

[one of the municipality participants reacted,] “what do you do, this is the area of health! Paying nurses and cars, it should come from the Obligatory Health Insurance Fund, and not from the MSSL.” And then B [a Ministry department director] says: “And what is the fault of that wee person that some ministries cannot achieve agreement, or one does not see a need [for home care]? Therefore we will do it nevertheless, and we’ll provide them [the people] the full help: not only social, but also medical.” (1st informant of macro level)

Ironically, to achieve collaborative discussions about developing IHC at the local level, the minister had to order it from above. The research team concluded that the changes in dynamics probably would not have occurred without the hands-on, catalytic assistance of advocates from the Ministry who worked at both the top level of the bureaucracy and at the service delivery levels. For once an order led to creativity rather than stagnation. Developing a clear understanding of aims and the example of the person-centered attitude were the uniting factors for collaboration and communication. This clarity would not have come without an effort to communicate in a new way, promoting dialogue. Thus, innovators were active on several levels.

### The innovation process: Using the “top” position to promote a new relationship of dialogue

The MSSL’s innovative social worker realized that not only was the idea of the innovation is important, but also how it was spread: she knew that in order to understand different contexts of people in different levels, it was very important to listen to those who worked in the field. The MSSL team made the decision that the creation of IHC will not be imposed in the familiar style of a top-down set of instructions. They were convinced of the crucial importance of implementing certain sub-processes under their overall partnership orientation. The team grew to collaboratively envision processes of initiating discussions and agreements with the administrators of municipality social service centres, promoting and elaborating the dialogue culture between personnel of all levels, encouraging initiative, and ensuring participation of participants in developing their own context-grounded models of IHC. Since the cultural practice had found people trying to enact what came from above, however feebly, it was thought to be imperative that the MSSL team showed and enacted the kind of honest participation that they were promoting. The innovators were also cautious about getting “correctly written” projects without any change happening. They tried to overcome this obstacle by finding people who would be interested.

“B asked: “But how should we do it so that the people do it not because of money, but because they really want to help patients and their caregivers?” And then we thought of a letter inviting not everybody, but those who have the initiative. At that point we also suppressed the information about the funds available.” (2nd informant of macro level)

In order to identify, promote and maintain the initiative in the municipal level, the innovators in the ministry continued with formulating a locally relevant, locally focused idea of IHC and looking for proactive municipalities (“early adopters”) simultaneously. It came out that people who recognised the need for home care nursing services, and already had some experience in looking for solutions of the problem and meeting the obstacles, responded.

The invitation, not a directive, for local professionals to show interest was unprecedented. Twenty-two municipalities out of 60 showed interest. However, it did not mean that finding a good contact and creating trust was easy. The first meeting drew 80 participants, but it was too big for the kind of dialogue that was the goal. The MSSL team decided to divide the municipalities into regional groups, to visit them and to discuss their ideas of IHC in order to better demonstrate and implement the core idea. The MSSL team not only created a possibility for regional innovators to discuss together, but they also tried to create a safe atmosphere for discussions. This encouraged the development and appreciation of horizontal ties.

“It is interesting that [during the regional meetings] they start to listen to each other. Of course, I moderate, try to create a safe atmosphere… I remember, we arranged in site 3 that we finish at 2 pm. And otherwise in official meetings people want to finish as soon as possible. So I say, it’s time, now we end [the meeting]. [Besides,] It was July, extremely hot. And nobody moves, they sit further on, continue to ask and discuss: “but wait, how will we do this? and that?” On the one side, in a monologue culture it looks like chaos; but in the discussion [culture] – this is the real work, they really think about reality, and not only for looks, for reports.” (2nd informant of macro level)

One of the ways to reduce competition and encourage collaboration was focusing on broader aims and diminishing competition by ensuring that all the participating sites would get the resources for their projects. For that, a new form of financing (state planning) was used to distribute the Program funds from the European Social Fund: instead of choosing the projects fitting the requirements best, the MSSL officials assured that all the participants who showed initiative and put effort would get the financing as a sort of *quid pro quo* for them offering their ideas in matters important to the state.

The invitation to write pilot projects allowed variety. Each site proposed its own project concerning the implementation of the IHC Program. These proposals reflected the specific local context of needs, resources, and existing ideas about the nature of care.

Understanding that there were a lot of expectations for the macro level officials, the innovators used their power position as an asset. They invited a dialogue and got a response. That the meetings took place in the regions and not in the capital/ministry was a sign of how important it is to pay attention to the local people and their conditions. Besides, it required skill to create a safe atmosphere: to reach that everyone feels heard, and to keep focused on the broader aim in order not to fight. Even everyone sitting around one table was unusual. The research team discussed this phenomenon repeatedly and at length because so much of the data revolved around it. As the data came in week by week from different sites, the researchers became persuaded that the innovation of IHC was possible because of the process, the way it was implemented.

### The innovative aspects of the services

There were several innovative aspects of the IHC service. First, medical and social areas were finally integrated, with both social and medical parts of the team paid from one source, and being one team. Second, the care was patient-centred. The services were allocated and changed, teams shared work-load and equipment based on the situations of the patients. Third, the help was professional. For example, in spite of having learned a lot about nursing, many relatives mentioned that they did not manage to treat bedsores; this changed with the nursing specialists starting to work. Fourth, the nursing services reached patients and their relatives at home. Fifth, it was really team-work, with having regular team meetings, with each member being important and discussing cases and participating in planning and providing service. As a team, they reacted to new situations, communicated with the family members and shared with them the care of the patients. The new opportunity to adapt services was used. For example, in some settings, the initial idea of visiting homes with mobile teams changed. Team members visited according to the plan of care, not in a set squad that visited everyone. Sixth, new positions of nursing assistant and kinesiotherapist were used for providing nursing at home.

Upon further assessment, the research team realized that there were many smaller innovations nested together under the broader idea of introducing IHC. For example, pilot sites were encouraged to do things according to different local conditions, and they did, leading to a range of new practices. New ways of doing nursing were evident [20]. New relationships among the local care authorities were significantly important in their own right. The current article reports on some of the big changes, paralleling many of Smale’s points, many of these local practices are worthy of their own study.

## Key people

Smale points out that without participation of key people there is no innovation. The findings of the current study are consistent with this idea. Key people have to be able to acquire new knowledge and skills in the process of adopting innovations through constant negotiation. The big part of success of the ICH creation process depended on certain pre-existing competences, i.e. attitudes, knowledge and skills that the participants in the innovation possessed, and on their ability to learn new competencies.

### The features of the IHC participants

Everyone in the project, researchers and workers alike, knew how the old system worked, and they worked within it. Many bureaucrats and professionals who had become established prior to independence seemed incapable of taking on new perspectives and ways of seeing and doing things. In contrast, the IHC innovators in the study were able to expand their ways of thinking from the dualistic, rule-bound, right or wrong mindset. It was as if they were waiting, even longing for a way to do things better. Despite the widespread futility and hopelessness, the innovators tapped personal and group assets that were open to new and better practices. The MSSL innovators were characterized by openness, partnership and honest dialogue to address problems.

An important element of the innovation was a visionary expert who guided but clearly did not control the process. An innovative social worker took the responsibility to move the idea of IHC forwards. The innovator’s knowledge of both medical and social areas and skill to create a safe atmosphere were crucial in bringing about change. Much of her work was done while enacting the processes that she was promoting. The research team found many examples of her ability not only to listen, but also to hear local practitioner perspectives, to find a common aim together, and to remain focused on it. The government that supported the MSSL innovators left the office at the end of 2012, but the skills and attitude of mind that she brought to the process were evident in data through the final data analyses in 2015.

A lot of participants of all levels were motivated by having personal experience related to the need of IHC. Many had experience with family members nursed at home. This personal experience seemed to offer a layer of motivation and skill that might have been hidden under the shell of the impassive bureaucrat. Many readily adopted a sensitive, caring, person-oriented attitude that was made legitimate in the discussion process and apparently became a source of emotional motivation. Moreover, a number of the municipalities’ administrators had the experience of establishing the earlier, very limited services of assisted care at home. Some even had tried to establish versions of IHC, albeit unsuccessfully. The previous experience helped to tackle the obstacles and to develop reality-based IHC projects. As some of the social workers additionally had an education in nursing, they were familiar with both medical and social systems and it helped to develop the integrated care models. Some familiarity with the Western systems of help at home, or other types of thinking, was also common.

The creation of a social innovation is always connected to risk. When the municipality participants expressed their wish to participate in the creation of pilot projects, it reflected their openness to initiative and risk. The researchers found that, unusually for the post-soviet context, the participants had the courage to make decisions, and dared to take risks. The majority of the participating sites’ municipality administrators mentioned that they liked to try out new things.

Summarizing, the people participating in the innovation were not afraid of changes, of non-standard and non-routine work, and of a new kind of relationship of dialogue with the uncertainty that it brings. Putting these qualities into action required a skilfully developed structure of sharing. Finding support and developing trust in each other, everyone was encouraged to take the initiative. It was obvious that all the participants who created and made IHC a reality became inspired, motivated and committed. Being alone, none of the participants could have achieved the reality of IHC, but together, with the conditions of dialogue provided, the change happened.

### Negotiating with clients

In general, the service receivers were deeply satisfied with their IHC experience. One service user, echoing many others called it a “revolution” in care, “the biggest step forwards through the whole time of regained Independence” [21]. However, there were also challenges of working at home that the IHC providers and their clients alike had to overcome. Service providers had to learn stronger ways to make contact and create a trust relationship with service recipients. In the home environment, the family members and the patient feel they are the hosts, meanwhile during the patient’s visits to healthcare agencies, the host institution supports the professional status of service providers. Inability to create trusting relationships is an obstacle to improving the quality of life of the patient.

For the clients who were invited to participate in the project, IHC services were also a social innovation that was not too easily accepted. Deeply rooted cultural practices and expectations may change slowly. Most of the clients/patients were of old age and chronically ill. Despite their current needs and even desires, their expectations were formed on another fundamental system of health and medicine. The patients needed time to get used to some new, unknown specialists visiting them at home. Besides, they felt it reduced their privacy. Furthermore, some respondents experienced discomfort with the idea that a stranger cares for your relative; the relatives were afraid of “what will the neighbours say”. In short, it came out that it was necessary to give time and effort for preparing both the service receivers and team members. Through communication, listening to the clients and their needs, and learning to work in teams, the tensions decreased, and overall patients and informal carers reactions to the IHC were extremely favourable.

## Discussion

Is it a surprise that a more contemporary, participatory innovation process led to fundamentally re-organized services that offer promises for greater effectiveness and for organizations that might be more capable of evolving? Or that the new ways of work mirrored some of the participatory methods of both the innovation and the research that documented it? On the surface, the answers would likely be “no” to both. However, for those familiar with the totalitarian historical context and the neo-totalitarian mindset that has persisted for 25 years since independence, the answer probably would be “yes”. Perhaps there is a generation of former soviet bureaucrats, some of whom have adapted, and many of whom have not adapted to independence and the norms of the new Europe, including the valuation of IHC. The study documents that the heart of the problem lies in large part with the people who persisted in the old ways of trying to maintain existing service delivery practices and to monitor services only in terms of their appearance of conforming to rules and directives.

The study documented the problems that came about in attempts at reform from the top level of ministries and from the field. Perhaps the early attempts to order integrated care were signs of change on the part of some government ministers. But clearly the policies did not affect the service delivery in the field in any constructive way. The study shows that these problems were results of discrepancies in understanding what was occurring in the field and how improvement might take place. Old ways of “care” of dependent people had been weakened through hospital closures, and the results could in fact persist indefinitely, or until policies and stable governments came about to let them develop in the interest of the people [22]. The importance of the findings lies in the documentation of a process in which a set of interactive, participatory methods did indeed lead to change. Several other elements were identified as well. The possibility of external funding of pilot projects should not be minimized. Not only was there money available for developing new services, but the European Union funders also provided a structure in their guidelines. This was used to help structure the innovation and particularly the role of action research as an integral part of the development. Proposals and administration of grant funds can be cumbersome, but using extramural funding structures positively as a way of legitimating innovations in otherwise indifferent settings should be recognized widely. It is critical that external influences are consistent with the principles that the innovators are attempting to establish. For example, using the case of mental disability [23] show how European Structural funds may serve to maintain large institutions over community care, and thereby prolong the soviet style marginalization and stigmatization of dependent people.

Two other findings are especially important. The relationship of key people at high levels was critical. A minister who was open to possibilities for improved services, the relationship between that minister and an informed expert innovator, and the ability of the innovator to work across levels of bureaucratic structure all the way down to the local service levels and even patients’ homes were relational keys to the success of the innovation. The second central finding was that while the social workers and nurses providing services often appeared to be ineffective and even indifferent to patients’ needs, in fact they held a great store of imagination, motivation, and energy to change. When the innovator was able to change the context to allow the safe expression and recognition of these attributes, desirable changes occurred. People began to work together.

A surprising finding was that new processes did indeed have to be ordered from above. But instead of the previous hollow policies, now there was follow-through. This is where the role of people who can span the levels of bureaucracy appears to be critical, especially in post-totalitarian settings.

The study findings are not easily generalizable in the conventional sense. However, they constitute a beginning in a region devoid of published accounts or evaluations of care [7]. Adequate funding for IHC after the pilot project finishes is uncertain, despite the national mandates. With a continuing financial commitment from government and additional development of IHC, it will become possible and necessary to evaluate the effectiveness of programs. It will be necessary to collect and analyze standardized data on effectiveness of home care and important outcomes such as patients’ mortality and morbidity, medication use and pain control, ability to conduct activities of daily living and mood [10,24,25,26]. Only when IHC is fully established can ongoing studies be designed to enhance service quality and to compare between models and countries. Government, academic, and practice personnel will have to continue cooperative relationships for either funding or evaluations to occur.

## Conclusion

Any kind of care reflects the society in which it takes place. Lithuania is still in transition from totalitarian communist society to a vibrant democracy, with social institutions oriented to the well-being of its citizens. Many individuals who functioned in the old system are still at work. Others who learned from them still have influence. Much of their behaviour is an expression of deeply imprinted attitudes of mind that serve to maintain old values of social control, rigid hierarchical organization, and devaluation of human dignity. Advocates of IHC that enacts opposing values have struggled in the shadow of this backdrop.

This study shows that innovators can be successful in reforming practices and making IHC real by understanding key dimensions of innovation, finding people who represent cracks in the authoritarian wall, strategically using external support, and modelling partnership across levels of bureaucracy. The key finding of the study in the context of societal transition is that the will to practice sound IHC was present or latent in many practitioners who had become relatively passive agents of the state, and that they were very open to participatory service development. The role of the innovators can be seen as a way to institute IHC. The role is also the embodiment of the social transition that has been so long coming.

## References

[1] Davis Ch, Kutzin J, Cashin Ch, Jakab M (2010). Understanding the legacy: health financing systems in the USSR 25 and central and eastern Europe prior to transition. Implementing Health Financing Reforms: Lessons from countries in transition.

[2] Gudžinskas L (2012). Lietuvos ir Estijos sveikatos apsaugos raida: panašios sąlygos, skirtingi rezultatai (Development of healthcare in Lithuania and Estonia: similar conditions, different results). Politologija.

[3] Žalimienė L, Lazutka R (2009). Socialinės globos paslaugos Lietuvoje: nuo valstybinio hierarchinio link mišrios ekonomikos modelio (Social care services in Lithuania: from hierarchical state model towards a mixed economy model). Pinigų studijos.

[4] LR Socialinės ir darbo apsaugos ministro įsakymas (1998). “Dėl socialinių paslaugų namuose plėtojimo krypčių ir stacionarių globos įstaigų darbo efektyvumo didinimo nuostatų patvirtinimo” (Order of Minister of Ministry of Social Security and Labour of Lithuanian Republic “Regarding directions of home care development and effectiveness of residential care).

[5] LR Sveikatos apsaugos ministro ir LR Socialinės ir darbo apsaugos ministro įsakymas (2007). “Dėl slaugos ir socialinių paslaugų bendro teikimo tvarkos aprašo patvirtinimo” (Order of Minister of Health of Lithuanian Republic and Minister of Ministry of Social Security and Labour “Regarding Description of Procedures of Common Nursing and Social Care Services Delivery).

[6] LR Socialinės apsaugos ir darbo ministro įsakymas dėl Integralios pagalbos plėtros programos patvirtinimo (2012). Order of Minister for Social Security and Labour Regarding the Approval of the Program of Integrated Care Development.

[7] Genet N, Boerma WG, Kringos DS, Bouman A (2011). Home care in Europe: a systematic literature review. BMC Health Services Research.

[8] Marcinowicz L, Chlabicz S, Konstantynowicz J, Gugnowski Z (2009). Involvement of family nurses in home visits during an 8-year period encompassing primary healthcare reforms in Poland. Health & Social Care in the Community.

[9] Kogoj A (2008). Care for people with dementia in Slovenia. Psychiatria Danubina.

[10] Bos JT, Frijters DHM, Wagner C, Carpenter I, Finne-Soveri H, Topinkova E (2007). Variations in quality of home care between sites across Europe, as measured by Home Care Quality Indicators. Aging clinical and experimental research.

[11] Argyris Ch, Schon D (1989). Participatory action research and action science compared: A commentary. The American Behavioral Scientist.

[12] Stake E, Denzin NK, Lincoln YS (2008). Qualitative Case Studies. Strategies of Qualitative Inquiry.

[13] Pettigrew A, Whipp R (1998). The Dimensions of Strategic Change. Managing Change for Competitive Success.

[14] Smale G (1998). Managing Change through Innovation.

[15] Smale G, Tuson G, Statham D (2005). Social work and social problems: working towards social inclusion and social change.

[16] Freire P (2005). Education for Critical Consciousness.

[17] Liamputtong P (2007). Qualitative Research Methods.

[18] Parahoo K (2006). Nursing Research. Principles, Process and Issues.

[19] LR Aukščiausiosios tarybos nutarimas (1991). “Dėl Lietuvos nacionalinės sveikatos koncepcijos ir jos įgyvendinimo [LR Supreme Council Resolution regarding National Health Care Concept and its Implementation.

[20] Danusevičienė L, Jurkuvienė R, Butkevičienė R, Gajdosikienė I (2014). The Changing Role of a Nurse in Lithuania related to Integrated Team-Based Home Care Pilot Projects. LSMU: Nursing Education, Research and Practice.

[21] Zailskaitė D (2014). Slaugos ir socialinės pagalbos projektas, deja, baigiasi. Kas laukia pacientų? (The project of nursing and social care. It’s a pity it is going to finish. What will happen with patients?). http://lsveikata.lt/medicinos-lyderiai/slaugos-ir-socialines-pagalbos-projektas-deja-baigiasi-kas-laukia-pacientu-1224.

[22] Navarro V, Muntaner C, Borrell C (2006). Politics and health outcomes. The Lancet.

[23] Puras D, Sumskiene E, Adomaityte-Subaciene I (2013). Challenges of prolonged transition from totalitarian system to liberal democracy. Journal of Social Policy & Social Work in Transition.

[24] Steffansson M, Pulliainen M, Kettunen A, Linnosmaa I, Halonen M (2016). The Association between Freedom of Choice and Effectiveness of Home Care Services. International Journal of Integrated Care.

[25] Sorbye LW, Garms-Homolova V, Henrard JC, Jónsson PV, Fialova D, Topinkova E, Gambass G (2009). Shaping home care in Europe: The contribution of the Aged in Home Care project. Maturitas.

[26] Foebel AD, van Hout HP, van der Roest HG, Topinkova E, Garms-Homolova V, Frijters D, Finne-Soveri H, Jónsson PV, Hirdes JP, Bernabei R, Onder G (2015). Quality of care in European home care programs using the second generation interRAI Home Care Quality Indicators (HCQIs). BMC Geriatrics.

